# Imperfect Linkage Disequilibrium Generates Phantom Epistasis (& Perils of Big Data)

**DOI:** 10.1534/g3.119.400101

**Published:** 2019-03-21

**Authors:** Gustavo de los Campos, Daniel Alberto Sorensen, Miguel Angel Toro

**Affiliations:** * Epidemiology & Biostatistics, Statistics & Probability departments, IQ-Institute for Quantitative Health Science and Engineering, Michigan State University, East Lansing, US; † Department of Molecular Biology and Genetics, Faculty of Science and Technology, Aarhus University, Aarhus, Denmark; ‡ Producción Animal, Universidad Politécnica de Madrid, Madrid, Spain

**Keywords:** epistasis, apparent epistasis, phantom epistasis, GWAS, linkage disequilibrium, imperfect LD, missing heritability, Big Data

## Abstract

The genetic architecture of complex human traits and diseases is affected by large number of possibly interacting genes, but detecting epistatic interactions can be challenging. In the last decade, several studies have alluded to problems that linkage disequilibrium can create when testing for epistatic interactions between DNA markers. However, these problems have not been formalized nor have their consequences been quantified in a precise manner. Here we use a conceptually simple three locus model involving a causal locus and two markers to show that imperfect LD can generate the illusion of epistasis, even when the underlying genetic architecture is purely additive. We describe necessary conditions for such “*phantom epistasis*” to emerge and quantify its relevance using simulations. Our empirical results demonstrate that phantom epistasis can be a very serious problem in GWAS studies (with rejection rates against the additive model greater than 0.28 for nominal p-values of 0.05, even when the model is purely additive). Some studies have sought to avoid this problem by only testing interactions between SNPs with R-sq. <0.1. We show that this threshold is not appropriate and demonstrate that the magnitude of the problem is even greater with large sample size, intermediate allele frequencies, and when the causal locus explains a large amount of phenotypic variance. We conclude that caution must be exercised when interpreting GWAS results derived from very large data sets showing strong evidence in support of epistatic interactions between markers.

A big challenge in genetics is to understand how variation at the DNA sequences translates into phenotypic variation. Genome-wide-association (GWA) studies address part of this challenge by testing for the association between phenotype (or a disease indicator) with genotype, one locus at a time. In the last decade, many GWA studies were conducted; these studies have reported thousands of SNPs (single nucleotide polymorphism) associated to complex traits and diseases (http://www.ebi.ac.uk/gwas).

Recently, several studies in model organisms (*e.g.*, Mackay 2014), humans (Strange, Ask, and Nielsen 2013) and agricultural species (*e.g.*, Huang, Xu, and Cai 2014), have used genotype data linked to phenotypes to investigate the presence of epistatic interactions between loci. Cordell (2002, 2009) and Wei, Hemani, and Haley (2014) provide comprehensive reviews of the methods commonly used to detect epistatic interactions.

There are several issues associated with studies aimed at detecting interactions, including matters of scale, the importance of the contribution of epistasis at the level of the genotype effects or at the level of the genotypic variance (*e.g.*, Hill, Goddard, and Visscher 2008) and how an interaction detected in a linear statistical model may be associated to biological pathways that underlie a complex trait (*e.g.*, Wang, Elston, and Zhu 2010; Aschard 2016). The latter becomes particularly problematic when the markers used to assess associations between SNPs and phenotypes (or a disease indicator) are in imperfect linkage disequilibrium (LD) with the alleles at the causal loci (*i.e.*, those responsible for inter-individual genetic differences in a trait or disease phenotype). Under those conditions, evidence supporting the existence of a non-null interaction between markers does not necessarily provide definite evidence of epistasis at causal loci. Specifically, when the SNPs used in association analyses are in imperfect LD with the alleles at causal loci, linear regression on SNPs may lead to unaccounted variance, or *missing heritability* (*e.g.*, Manolio *et al.* 2009; de Los Campos *et al.* 2015). When this unaccounted additive signal is correlated with interaction contrasts, the “illusion” of epistasis is created even for traits that are purely additive.

Several authors have expressed concerns about the role that LD can have on the detection of epistasis (Wood *et al.* 2014; W.-H. Wei, Hemani, and Haley 2014). However, these problems have not been quantified nor have they been given a precise mathematical treatment. In this study, we present a simple three locus model involving a causal (unobserved) locus and two markers that makes explicit how *phantom epistasis* may emerge even in systems that are strictly additive. We use this model to derive a set of conditions that are necessary for the occurrence of phantom epistasis, and quantify the magnitude of the problem using simulations based on real human genotypes from the UK-Biobank. The existence of phantom epistasis is also studied using extensions of the model that include dominance and multiple loci. Our results indicate that imperfect LD can lead to seriously inflated type-I error rates. We also show that the rate of detection of phantom epistatic interactions increases with sample size; this should be considered when testing for epistatic interactions using big data sets such as the ones that are becoming available.

## Materials and Methods

We begin by considering a simple model with three biallelic loci. One of them, denoted as $z_{i}$, represents a causal locus (also referred as to the ‘quantitative trait locus’, QTL) on observation *i* and has a direct effect on the expression of the phenotype $y_{i}$. The other two loci, denoted as $x_{1i}$ and $x_{2i}$, are markers that are possibly in LD with the QTL but have no causal effect on the phenotype. For SNPs, a standard practice is to code genotypes by counting at each of the loci the number of copies of a reference allele carried by the *i^th^* individual. Here, to facilitate the presentation we assume that genotypic codes and phenotypes are expressed as deviations from their corresponding means; therefore $E\left(z_{i}\right)=E\left(x_{1i}\right)=E\left(x_{2i}\right)=E\left(y_{i}\right)=0$. In this setting, a single locus strictly additive model takes the form

$$y_{i}=z_{i}b+\delta_{i},$$ [1]

where b is the additive effect of an allele substitution at locus z, and $\delta_{i}$ is an error term. Evidently, with only one causal locus there is no epistasis. Next, suppose that an instrumental regression of the form

$$y_{i}=x_{1i}\beta_{1}+x_{2i}\beta_{2}+x_{1i}x_{2i}\beta_{12}+\varepsilon_{i}$$ [2]

is used to investigate the presence of epistasis. Here, the $\beta^{’}s$ are regression coefficients that are functions of the QTL effect (*b*) and of the (multilocus) LD involving the two markers and the QTL genotypes. In the population, given the centered genotype codes, the regression coefficients entering in the right-hand-side of [2] are

$$\left[\begin{array}{c} \beta_{1}\\ \beta_{2}\\ \beta_{12} \end{array}\right]={\left[\begin{array}{ccc} E\left(x_{1i}^{2}\right) & E\left(x_{1i}x_{2i}\right) & E\left(x_{1i}^{2}x_{2i}\right)\\ E\left(x_{1i}x_{2i}\right) & E\left(x_{2i}^{2}\right) & E\left(x_{1i}x_{2i}^{2}\right)\\ E\left(x_{1i}^{2}x_{2i}^{}\right) & E\left(x_{1i}x_{2i}^{2}\right) & E\left(x_{1i}^{2}x_{2i}^{2}\right)-E{\left(x_{1i}x_{2i}\right)}^{2} \end{array}\right]}^{-1}\times \left[\begin{array}{l} E\left(y_{i}x_{1i}\right)\\ E\left(y_{i}x_{2i}\right)\\ E\left(y_{i}x_{1i}x_{2i}\right) \end{array}\right].$$

If the random residual $\delta_{i}$ in expression [1] is orthogonal to the genotypes, then $E\left(y_{i}x_{1i}\right)=E\left(z_{i}x_{1i}\right)b$, $E\left(y_{i}x_{2i}\right)=E\left(z_{i}x_{2i}\right)b$ and $E\left(y_{i}x_{1i}x_{2i}\right)=E\left(z_{i}x_{1i}x_{2i}\right)b$. Thus, the population regression coefficients are defined by

$$\left[\begin{array}{c} \beta_{1}\\ \beta_{2}\\ \beta_{12} \end{array}\right]={\left[\begin{array}{ccc} E\left(x_{1i}^{2}\right) & E\left(x_{1i}x_{2i}\right) & E\left(x_{1i}^{2}x_{2i}\right)\\ E\left(x_{1i}x_{2i}\right) & E\left(x_{2i}^{2}\right) & E\left(x_{1i}x_{2i}^{2}\right)\\ E\left(x_{1i}^{2}x_{2i}^{}\right) & E\left(x_{1i}x_{2i}^{2}\right) & E\left(x_{1i}^{2}x_{2i}^{2}\right)-E{\left(x_{1i}x_{2i}\right)}^{2} \end{array}\right]}^{-1}\times \left[\begin{array}{l} E\left(z_{i}x_{1i}\right)\\ E\left(z_{i}x_{2i}\right)\\ E\left(z_{i}x_{1i}x_{2i}\right) \end{array}\right]b\;.$$ [3]

This indicates that the regression coefficients of the instrumental model [2] are not only functions of the QTL effect (*b*) and of pair-wise (1^st^ order) LD but also of higher order LD, *e.g.*, joint disequilibrium at three loci, $E\left(z_{i}x_{1i}x_{2i}\right)$. The moments involved in the right hand-side of [3] are diploid genotypic measurements of LD. Under random mating these genotypic measures of LD are equal to twice the standard haploid measures of LD (the *D*-coefficients for two and three loci linkage disequilibrium; see section 1 of the Supplementary Methods for further details).

In the population, the interaction effect $\beta_{12}$ is given by a linear combination involving two-loci LD between one of the markers and the QTL, and three-loci LD involving the two markers and the QTL: $\beta_{12}=\left[t_{31}E\left(z_{i}x_{1i}\right)+t_{32}E\left(z_{i}x_{2i}\right)+t_{33}E\left(z_{i}x_{1i}x_{2i}\right)\right]b$. Here, the $t^{’}s$ are the entries of the third row of the inverse of the coefficient matrix

$$T^{-1}={\left[\begin{array}{ccc} E\left(x_{1i}^{2}\right) & E\left(x_{1i}x_{2i}\right) & E\left(x_{1i}^{2}x_{2i}\right)\\ E\left(x_{1i}x_{2i}\right) & E\left(x_{2i}^{2}\right) & E\left(x_{1i}x_{2i}^{2}\right)\\ E\left(x_{1i}^{2}x_{2i}\right) & E\left(x_{1i}x_{2i}^{2}\right) & E\left(x_{1i}^{2}x_{2i}^{2}\right)-E{\left(x_{1i}x_{2i}\right)}^{2} \end{array}\right]}^{-1}$$

### Required conditions for phantom epistasis

If the QTL is in LE with the two markers, then $p\left(z_{i},x_{1i},x_{2i}\right)=p\left(z_{i}\right)p\left(x_{1i},x_{2i}\right)$. Consequently, $E\left(z_{i}x_{1i}\right)=E\left(x_{1i}\right)E\left(z_{i}\right)=0$, $E\left(z_{i}x_{2i}\right)=E\left(x_{2i}\right)E\left(z_{i}\right)=0$, and $E\left(z_{i}x_{1i}x_{2i}\right)=E\left(x_{1i}x_{2i}\right)E\left(z_{i}\right)=0$. Therefore, all elements of the right-hand-side of [3] are equal to zero and, thus $\beta_{1}=\beta_{2}=\beta_{12}=0$. Therefore, for phantom epistasis to emerge *a first necessary condition* is that *the QTL must be in LD with at least one of the SNPs*. (This condition is a special case of a more general condition that we discuss below.)

On the other extreme, if there is perfect LD between the QTL and the marker pair $\left(x_{1i}x_{2i}\right)$, then the QTL genotype can be expressed as a linear function of the two marker genotypes $z_{i}=x_{1i}\beta_{1}+x_{2i}\beta_{2}$. In this case, a linear regression on the two markers captures fully the QTL variance and therefore the interaction term will be equal to zero. (A derivation of this intuitive result is presented section 2 of the Supplementary Methods.) Therefore, perfect LD is a sufficient condition for $\beta_{12}=0$. Consequently, a *second necessary condition* for phantom epistasis to emerge is *imperfect LD between the QTL and the marker pair*. This guarantees that some fraction of the QTL variance is not captured by linear regression on the two marker genotypes. Furthermore, if the left-out QTL signal is not orthogonal to the interaction contrast $x_{1i}x_{2i}$, then $\beta_{12}\neq 0.$

Consider now an intermediate case where one of the markers (say $x_{2i}$) is independent of the pair formed by the QTL and the other marker $\left(z_{i},x_{1i}\right)$. This implies that $p\left(z_{i},x_{1i},x_{2i}\right)=p\left(z_{i},x_{1i}\right)p\left(x_{2i}\right)$. Under this condition, because the two markers are in LE, the coefficient matrix and its inverse ($T^{-1}$) is diagonal; therefore, $\beta_{12}=\frac{E\left(z_{i}x_{1i}x_{2i}\right)}{E\left(x_{1i}^{2}x_{2i}^{2}\right)-E{\left(x_{1i}x_{2i}\right)}^{2}}b$. Moreover, $E\left(z_{i}x_{1i}x_{2i}\right)=E\left(z_{i}x_{1i}\right)E\left(x_{2i}\right)=0$, implying that $\beta_{12}=0$. Therefore, a *third necessary condition* for phantom epistasis to emerge is that *the three loci must be jointly in LD*.

***In conclusion*,** in the system discussed above, *phantom epistasis can emerge if the three loci are in mutual but imperfect LD*. Unfortunately, this condition cannot be assessed when the QTL genotype is unknown. Empirically only LD between the two markers can be assessed.

### Phantom epistasis in multi-locus models

In an additive multi-locus model expression [1] becomes $y_{i}=\overset{q}{\underset{j=1}{\sum }}z_{ij}b_{j}+\delta_{i}.$ When testing pairwise interactions between markers the empirical model [2] remains unchanged; therefore, if the two markers involved in the instrumental model are in LE the matrix representing left-hand side of the OLS systems of equations is diagonal. In this setting, phantom epistasis emerges when the third right-hand side term, $E\left(x_{1i}x_{2i}\overset{q}{\underset{j=1}{\sum }}z_{ij}b_{j}\right)$, is nonzero. Since the expectation is a linear operator, $E(x_{1i}x_{2i}\overset{q}{\underset{j=1}{\sum }}z_{ij}b_{j}(=\overset{q}{\underset{j=1}{\sum }}b_{j}E\left(x_{1i}x_{2i}z_{ij}\right)$. If one of the markers is in LE with the other marker and with all the QTL then $E\left(x_{1i}x_{2i}z_{ij}\right)=0\;\forall \;j$, thus there will not be phantom epistasis.

***In the presence of dominance*,** the causal (single locus) model becomes $y_{i}=az_{i}+dz_{i}^{2}+\delta_{i}\;$ where a and d are additive and dominance values, respectively. If the empirical model of expression [2] is used to test for epistatic interactions then the left-hand-side of expression [3] remains unchanged, but the right-hand-side becomes

$$\left[\left(\begin{array}{c} E\left(x_{1i}z_{i}\right)\\ E\left(x_{2i}z_{i}\right)\\ E\left(x_{1i}x_{2i}z_{i}\right) \end{array}\right)a+\left(\begin{array}{c} E\left(x_{1i}z_{i}^{2}\right)\\ E\left(x_{2i}z_{i}^{2}\right)\\ E\left(x_{1i}x_{2i}z_{i}^{2}\right) \end{array}\right)d\right]$$

indicating that both dominance and additive effects can indeed contribute to phantom epistasis. In this system, phantom epistasis will emerge whenever $E\left(x_{1i}x_{2i}z_{i}\right)a+E\left(x_{1i}x_{2i}z_{i}^{2}\right)d\neq 0.$ The conditions needed for phantom epistasis to emerge in a system involving dominance are the same as the ones described for the additive model. These include, first, imperfect LD between $z_{i}$ and $z_{i}^{2}$ with the marker pair $\left(x_{1i},x_{2i}\right)$ such that either $z_{i}$ or $z_{i}^{2}$ or both cannot be fully explained by a linear combination of the two markers. Second, phantom epistasis requires mutual LD at the three loci. If one of the markers (say $x_{2i}$) is independent of the other-marker-QTL pair, then, $E\left(x_{1i}x_{2i}z_{i}\right)a+E\left(x_{1i}x_{2i}z_{i}^{2}\right)d=E\left(x_{2i}\right)\left[E\left(x_{1i}z_{i}\right)a+E\left(x_{1i}z_{i}^{2}\right)d\right]=0$.

### Simulation studies

The analytical results presented in the previous section indicate that multi-locus LD plays an important role in determining whether phantom epistasis may emerge. To shed light on the nature and the magnitude of the problem we conducted Monte Carlo simulations using real human genotypes of distantly related white Caucasian individuals from the UK-Biobank.

In a ***first set of simulation scenarios***, we generated data according to an additive model with a single causal locus (as in [1]) that explained either 0.5 or 1% of the phenotypic variance. The position of the QTL genotype $z_{i}$ was determined by randomly choosing a marker position on human chromosome 1; marker $x_{1i}$ was always adjacent (“to the left”) to the QTL whereas the other marker ($x_{2i}$) was placed at increasing base pair distance (“to the right”) of the QTL.

We analyzed the simulated phenotypes using an instrumental model such as the one in [2] extended with inclusion of an intercept and the top 5 SNP-derived PCs to avoid confounding due to any substructure that may be present (see section 3 of Supplementary Methods for details). The null hypothesis $\left(H_{0}:\beta_{12}=0\right)$ was rejected at a 0.05 significance level; therefore, rejection rates over 0.05 were interpreted as indicative of phantom epistasis. All analyses were done with R (Rcore development team (2012) using the BGData R-package (Grueneberg and de los Campos, 2019).

Since the power to detect a non-null interaction effect depends on sample size we analyzed data using a sample sizes of n = 10K, 50K, 100K and 250K (K = 1,000).

### Alternative simulation scenarios

#### SNPs in different chromosomes:

To assess the potential impact of long-range LD, in a new analysis setting we maintained the QTL-proximal-marker pair ($x_{1i},z_{i}$) in chromosome 1 as in our main simulation scenario but now positioned the distal marker ($x_{2i}$) in a randomly chosen position on chromosome 2. In this data set, we do not expect high levels of LD between SNPs in different chromosomes; however, at least in theory, this could happen if selection favors combinations of alleles, thus inducing LD at physically unlinked loci (Bulmer 1971).

#### Multi-locus models:

To consider the problem of phantom epistasis in multi-locus models we first assumed that the genetic architecture of the trait was determined by a major QTL plus an infinitesimal effect that was strictly additive. The trait heritability was 0.5, the main QTL explained 1% of the variance and the infinitesimal component the remaining 49% (see Section 3 of the Supplementary Methods for further details).

Second, we simulated data using a model that contains three marker-QTL pairs: $\left(x_{1i},z_{1i}\right)$, $\left(x_{2i},z_{2i}\right)\;$and $\left(x_{3i},z_{3i}\right)$. As before, the trait was strictly additive with the genetic effect, $\overset{3}{\underset{j=1}{\sum }}z_{ij}b_{j}$, explaining 1% of the phenotypic variance (each QTL explained 1/3 of the total genetic variance). The three pairs were in chromosome 1. Within a pair, the marker was the SNP immediately adjacent to the QTL in the array. To study the effects of LD we moved pairs 1 and 3 further apart and always maintained the 2^nd^ pair in the middle point (based on base-pair distance). The empirical model used to test for interaction was as that in expression [2] with $x_{3i}$ in place of $x_{2i}$; that is we tested for phantom epistasis between SNPs in the first and third pair. This scenario aimed at describing problems that may emerge due to an unaccounted QTL ($z_{2i}$ in this case) which may be in simultaneous LD between the two SNPs involved in the interaction.

### Data availability

The genotypes used in the simulation were from the UK Biobank. Data were acquired under project identification number 15326. The data are available for all bonafide researchers and can be obtained by applying at http://www.ukbiobank.ac.uk/register-apply/. The Institutional Review Board (IRB) of Michigan State University has approved this research with the IRB number 15-745. Supplemental material available at Figshare: https://doi.org/10.25387/g3.7848233.

## Results

We begin presenting results from our main simulation scenario which involves a single-locus with two SNPs, one proximal ($x_{1i})$ and one distal ($x_{2i})$ to the QTL.

**Figure 1:**
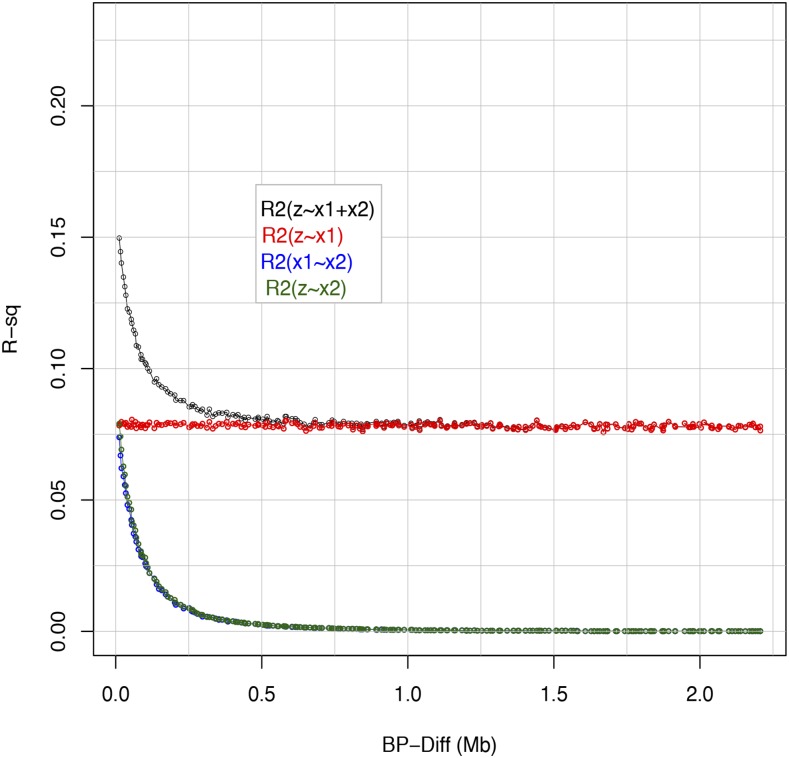
Average R-squared between pairs of loci and proportion of variance of the QTL genotype explained by the two markers, $R^{2}\left(z_{i}∼x_{1i}+x_{2i}\right)$, *vs.* distance between the QTL ($z_{i}$) and the distal marker ($x_{2i}$). Marker $x_{1i}$ was always adjacent to the QTL.

Figure 1 shows measures of linkage disequilibrium between the three loci $\left(z_{i},x_{1i},x_{2i}\right)\;$involved in the system. The average (across Monte Carlo replicates) proportion of variance of the QTL ($z_{i}$) explained by the most adjacent marker ($x_{1i}$) was about 0.085; however, the distribution of this statistic is highly skewed. When $x_{1i}$ and $x_{2i}$ were the two flanking markers of the QTL, on average they jointly explained 15% of the QTL variance. Therefore, on average there was a sizable rate of “missing” heritability. The R-sq. between $x_{2i}$ and either the other marker or the QTL, falls very quickly for lags between 0-0.5Mb and reached near zero values at approximately 1 Mb (Figure 1).

**Figure 2:**
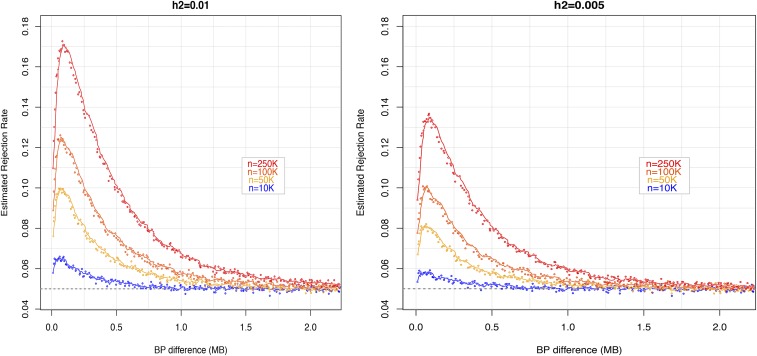
Empirical rejection rates *vs.* distance between the QTL and the distal marker, by proportion of variance explained by the QTL (left and right panels) and sample size (curves). In the simulations, a single QTL ($z_{i}$) had an additive effect that explained either 1% (left) or 0.5% (right) of the phenotypic variance. The empirical model considered two SNPs with no causal effect. One of them ($x_{1i}$) was adjacent to the QTL and the other one ($x_{2i}$) was placed at increasing distance from the pair ($x_{1i},z_{i}$). Rejection of the null hypothesis (no interaction between $x_{1i}$ and $x_{2i})\;$was conducted at a 0.05 significance level. Empirical rejection rates above 0.05 are indicative of phantom epistasis.

Figure 2 displays empirical rates of rejection by BP-distance between the QTL and the distal marker ($x_{2i}$), by sample size and by proportion of variance explained by the QTL. In absence of phantom epistasis, the empirical rejection rate should be very close to the significance level (0.05). For the largest sample size, the curve relating empirical rejection rates with BP distance was clearly above 0.05 for distances of up to 2MB. In all the scenarios, the highest rejection rates were observed when $x_{2i}$ and the QTL were at a distance of about 0.15 MB; here the empirical rejection rate was ∼0.13 when the QTL explained 0.5% of the phenotypic variance and as high as 0.17 when the QTL explained 1% of the variance. The latter (0.17) is more than three times the nominal rate of rejection expected under the absence of phantom epistasis (0.05). The curves relating empirical rejection rates with physical distance reach the nominal rejection rate of 0.05 at ∼1Mb for n = 10,000; however, for larger sample size the curves stayed above 0.05 even for distances longer than 1Mb.

**Figure 3:**
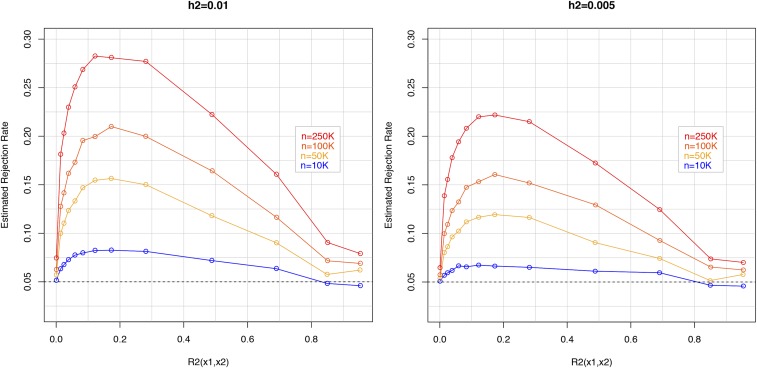
Empirical rejection rates *vs.* R-squared between the proximal and distal marker, by proportion of variance explained by the QTL (left and right panels) and sample size (curves). The simulation setting here was the same as that in Figure 2: a single QTL ($z_{i}$) had an additive effect that explained either 1% (left) or 0.5% (right) of the phenotypic variance. The empirical model considered two SNPs with no causal effect. One of them ($x_{1i}$) was adjacent to the QTL and the other one ($x_{2i}$) was placed at increasing distance from the pair ($x_{1i},z_{i}$). Rejection of the null hypothesis (no interaction between $x_{1i}$ and $x_{2i})\;$was conducted at a 0.05 significance level. Empirical rejection rates above 0.05 are indicative of phantom epistasis.

Figure 3 displays another way of viewing the simulation results of Figure 2 where the average rejection rate is calculated within bins of R-sq. between the two markers. When the two markers were uncorrelated, rejection rates were very close to 0.05 indicating absence of phantom epistasis. However very small LD between the two markers generates considerably higher rejection rates: an $R^{2}\left(x_{1i},x_{2i}\right)∼0.1$ leads to rejection rates as high as 0.28 with the largest sample size when the proportion of variance explained by the QTL was 1%. The maximum rejection rates occur when the R-sq. between markers is between 0.1 to 0.2. Beyond this value in the range (0.2-0.9) rejection rates follow a linear decline.

**Figure 4:**
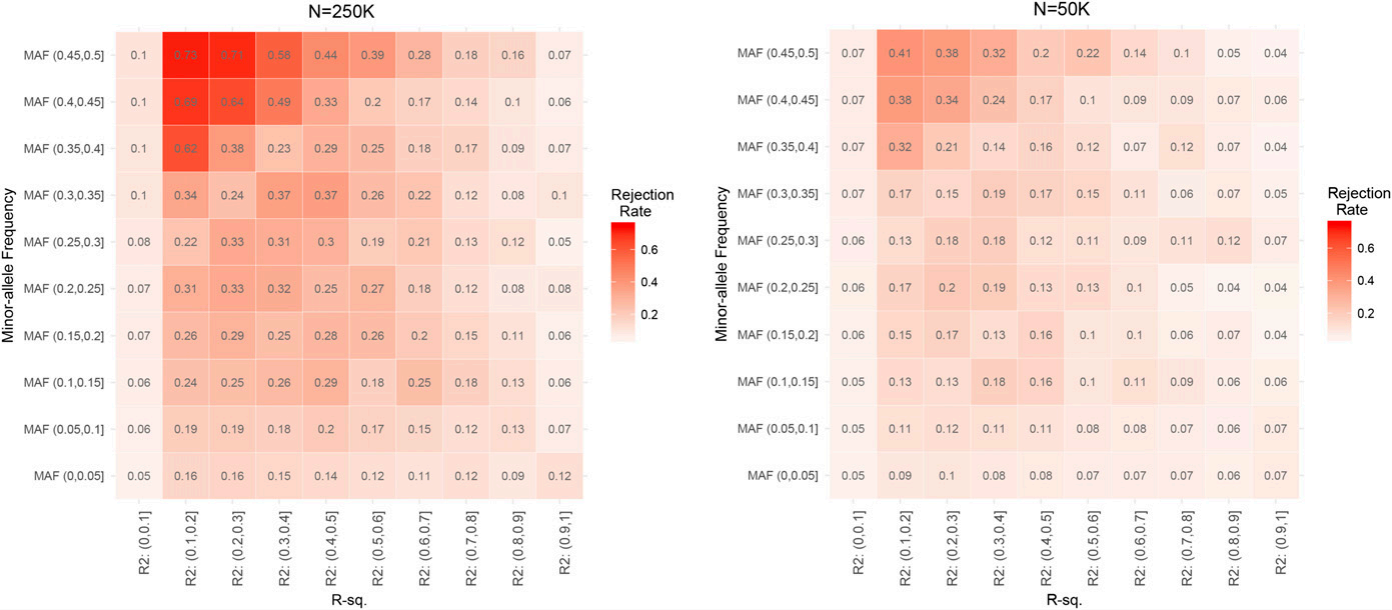
Heatmap of empirical rejection rates by sample size (left and right panels), minor allele frequency (average of the two SNPs) and R-squared between the two SNPs involved in the interaction. The simulation setting here was the same as the one used to produce the results of Figures 2 and 3. The results in the figure correspond to an additive QTL that explained 1% of the variance and a sample size of either 250K (left) or 50K (right).

Finally, Figure 4 shows the empirical rejection (for the scenario where the QTL explained 1% of the phenotypic variance) rates by minor-allele frequency and the R-squared between the two markers involved in the interaction. For any given bin of minor-allele frequency, the rejection rate was close to 0.05 when R-squared was close to zero, it increases reaching a maximum for R-squared values of 0.1-0.3, and then shows a decline reaching again values close to 0.05 when R-squared is larger than 0.9. These patterns are the same as the ones displayed in Figure 3. Interestingly, within a bin of R-squared, when phantom epistasis existed (*i.e.*, when R-squared was neither null nor perfect) rejection rates were maximum for intermediate allele frequencies and decreased with extreme allele frequencies. This is likely a consequence of the fact that intermediate allele frequencies confer higher power to the test.

The results from the model with ***SNPs in different chromosomes*** are provided in Table S1. In all cases, the rejection rates were very close to 0.05 suggesting that phantom epistasis between SNPs on different chromosomes, if it exists, it occurs at a rate that does not induce an inflation of rejection rates above the significance level.

### Multi-locus models

Figure S1 summarizes the results from the simulation scenarios that included an ***infinitesimal effect***. The empirical rejection rates were almost identical to those obtained, for the same sample size, with the single-QTL model (compare Figure S1 with the curves for N = 50K and 250K in the left panel of Figure 2). This suggests that the interaction contrasts were quasi orthogonal to the infinitesimal effect, which is probably a consequence of the short span of LD in this population.

The results from a scenario involving ***three marker-QTL pairs*** are summarized in Figure S2. The rates of rejection in this simulation scenario were very similar to the ones observed in the single QTL model (compare Figure S2 with curves corresponding to N-50K and N = 250K in the left panel of Figure 2) with mildly elevated rejection rates when the distance between pairs 1 and 3 was between 0.1-1Mb, due to the non-zero term $b_{2}E\left(x_{1i}x_{3i}z_{2i}\right)$ of the right-hand-side of [3], that causes $\beta_{12}\neq 0$. However, as the distance between the markers involved in the interaction (*i.e.*, the 1^st^ and 3^rd^ marker) increased, the estimated rejection rates approached the nominal rejection rate. Specifically, when the distance between the 1^st^ and the 2^nd^ pair is of 2Mb or larger the three pairs become independent and phantom epistasis vanishes.

## Discussion

There is a substantial amount of literature reporting the presence of epistasis affecting complex traits but results, when scrutinized, have been controversial. Sometimes the controversy spawns from the suspicion that epistatic interactions may be capturing additive signals that were missed by the baseline additive model used to test interactions. For instance, Hemani *et al.* (2014) identified 30 pairs of SNPs that interact influencing gene expression and that were replicated across two independent studies. In a subsequent study (Wood *et al.* 2014) replicated many of the interactions reported by Hemani *et al.*; however, in each case, using sequence data, a single third variant could explain all the apparent epistasis. This happened even after removal of all pairs of SNPs with $r^{2}<0.1$ which was suggested by Wei, Hemani, and Haley (2014) as a means to minimize confounding due to haplotype effects.

However, the problem of why and under what conditions additive effects may generate “epistatic signals” has not be formalized. In this work, we considered a simple three locus model to reveal conditions that lead to phantom epistasis. We show that phantom epistasis emerges in the presence of simultaneous but imperfect mutual LD between the three loci (the QTL and the two markers involved in the interaction). This conceptually simple three loci model can be extended to more complex settings (*e.g.*, multiple QTL-marker pairs) without affecting the underlying source of the principle: if additive QTL variance is imperfectly captured by linear regression on markers and the unexplained variation is not orthogonal to interaction contrasts, then phantom epistasis emerges.

We stress that simultaneous LD between the triplet $\left(x_{1i},x_{2i},z_{i}\right)\;$is required for phantom epistasis to emerge. At least in theory, it is possible to have cases where the two SNPs involved in an interaction are in LE yet, they are jointly in LD with an unobserved QTL. An example featuring such patterns is discussed by Wei *et al.* (2013) who present a model with dominance at a causal locus that generates phantom epistasis between two flanking markers that are marginally independent. In Wei’s example the two SNPs are jointly in LD with the QTL and therefore, $E\left(x_{1i}x_{2i}z_{i}\right)$ and $E\left(x_{1i}x_{2i}z_{i}^{2}\right)$ are nonzero, explaining why the model generates phantom epistasis. On the other hand, if one of the markers, *e.g.*, $x_{1}$, is independent of the remaining pair $\left(x_{2i},z_{i}\right),$ then $E\left(x_{1i}\;x_{2i}z_{i}\right)=E\left(x_{1i}\right)E\left(x_{2i}z_{i}\right)=0$ and phantom epistasis does not not emerge.

### Inferences Under imperfect LD

Several authors (M E Goddard 2009, de los Campos *et al.* 2013; de Los Campos, Sorensen, and Gianola 2015; Gianola *et al.* 2015) have studied the role of imperfect LD on related inferential problems, including missing heritability and whether imperfect LD can lead to estimates of genomic correlations between traits that are different than the underlying genetic correlations (Gianola *et al.* 2015). In all these cases, imperfect LD generates inferential difficulties; phantom epistasis is another inferential problem arising when the markers used for inferences are in imperfect LD with causal variants.

Testing interactions among weakly correlated SNPs only (*e.g.*, considering only SNP-pairs with $r^{2}<0.1)$ is not a solution: indeed, our simulation results show that weak LD between markers (*e.g.*, R-sq. between 0.05 and 0.1) can lead to large numbers of false discoveries especially when sample size is large. However, our simulations also show that near independence between the two SNPs (*e.g.*, R-sq. <0.01), a condition that in the data set used in this study was achieved at distances of 1.5Mb-2Mb or longer, was enough in the scenarios tested to guarantee rejection rates very close to the significance level.

### Perils of Big Data

If phantom epistasis exists (*i.e.*, if the population coefficient $\beta_{12}\neq 0$) whether it is detected or not depends on the power of the study which increases with sample size. Our simulation results demonstrate this clearly: pairwise R-sq. of 0.1 between markers and large sample size (*e.g.*, n > 100K) generates clear signs of phantom epistasis. However, rejection rates are not highly elevated over the significance level when sample size was smaller (n = 10k) because at that R-sq. the size of the interaction effect is small and therefore the power to detect such small interaction effect with small sample size is low. Big Data are a blessing for genomic analysis of complex traits; however, in some cases, large sample size can make an inferential problem even more problematic.

### The proportion of variance explained by the QTL plays a role similar to that of sample size

The larger the amount of variance explained by the QTL, the higher the power to detect phantom epistasis due to imperfect LD. To assess this, we repeated our simulation with a QTL that explained 50% of the phenotypic variance. The results (see Figure S3) showed, as one would expect, higher rejection rates than the one observed when the QTL explained a small fraction of the phenotypic variance (compare Figure 2 with Figure S3). Importantly, and in agreement with what our model predicts, the rejection rate reaches the significance level when the two SNPs become independent.

### Phantom Epistasis in multi-locus models

The presence of ***a polygenic effect*** in our simulations did not lead to notoriously inflated rejection rates compared with the ones detected with the single locus model suggesting that the interaction contrasts were quasi orthogonal to the infinitesimal effect. This is probably because LD in this population spans over very short distances and therefore it is highly unlikely to find a pair of markers that are strongly correlated with infinitesimal effects emerging from large numbers of loci distributed over the genome. On the other hand, local polygenicity (*i.e.*, the accumulation of many small-effect loci in a region with moderate or high LD) may lead to more serious apparent epistasis.

#### Local epistasis?

Several studies have reported results highlighting the importance of ‘local’ epistatic interactions (*e.g.*, Wei, Hemani, and Haley 2014; He *et al.* 2017). From a biological perspective, it is plausible that multiple mutations in a gene may have collectively a larger impact than the simple sum of the effects of each mutation individually. And this could manifest itself as “haplotype effects” (*e.g.*, Haig 2011). However, phantom epistasis provides an alternative explanation for why epistatic interactions detected in GWAS occur between loci that are physically close. Indeed, we show analytically and empirically that LD between SNPs is required for phantom epistasis to appear, thus, phantom epistasis is expected to be predominantly a ‘local’ phenomena.

#### Inflation of rejection rates due to incorrect statistical assumptions?

Based on an earlier version of this manuscript Dr. P. Visscher (personal communication) commented that under imperfect LD the distribution of the error terms of the empirical model is not normal. Indeed, because the QTL genotype has three levels, under imperfect LD the distribution of the error terms would be a mixture of three normal distributions with different means. This could certainly explain the inflation of rejection rates in small samples because the conditions for the test statistic to follow a *t* distribution are not met. However, in large samples, the test statistic follows a normal distribution even if errors are not Gaussian (this is simply an application of the Central Limit Theorem). Therefore, with the size of samples used in standard GWA studies, lack of normality of the error term should not be a cause for inflated rejection rates.

A second possible cause for inflated rejection rate of the null hypothesis can be underestimation of the error variance of the empirical model. However, it can be shown that the estimator of the error variance of the empirical model used in the simulations is unbiased with respect to the marginal distribution of the data. Therefore, phantom epistasis cannot be attributed to underestimation of the error variance.

### The additive-non-additive conundrum

Quantitative genetics studies properties of complex traits using regression analysis. In the field a careful distinction is made between observable and causal features of complex traits. For instance, it is well established that the linear regression of a phenotype on allele content yields estimates of the average effect of allele substitution and that both truly additive as well as dominance and epistatic effects can contribute to allele substitution effects. Furthermore, theoretical and empirical research has demonstrated that highly non-linear systems can generate signals that can often be explained almost completely with a linear model (Hill, Goddard, and Visscher 2008). For this reason, in general, one cannot make causal statements about gene action from observational variance component analyses (*e.g.*, W. Huang and T. F. C. Mackay 2016). Complicating matters even further we show in this study that the opposite can happen: under a purely additive model, imperfect LD can generate non-additive signals!

The recognition that phantom epistasis may be an important phenomenon does not negate the relevance of gene-gene interactions at the causal level. It simply stresses the difficulties that one faces when trying to learn about causal features of a system using observational data and inputs (markers) which are proxies for the underlying variants that may have causal effects on traits.

#### Phantom epistasis: an opportunity to improve predictive performance?

In this work, we have stressed that imperfect LD can limit the possibility to learn about causal effects. However, linear and non-linear genomic regressions can be very powerful predictive machines, and it is well established that the model that is best for inferences is not necessarily the best predictive tool. Phantom epistasis creates inferential problems but also opens opportunities for improving prediction models. Indeed, by capturing signals that are missed by an additive model, non-linear models using interactions between markers may increase the amount of genetic variance captured and improve prediction accuracy. This may explain, for instance why some non-linear models such as kernel regressions have shown better predictive performance than additive models, especially in breeding populations with long-span LD and low marker density (de los Campos *et al.* 2010).

## References

[bib1] Aschard H., 2016 A Perspective on Interaction Effects in Genetic Association Studies. Genet. Epidemiol. 40: 678–688. 10.1002/gepi.21989 27390122 PMC5132101

[bib3] Bulmer M. G., 1971 The Effect of Selection on Genetic Variability. Am. Nat. 105: 201–211. 10.1086/282718

[bib4] Cordell H. J., 2002 Epistasis: What It Means, What It Doesn’t Mean, and Statistical Methods to Detect It in Humans. Hum. Mol. Genet. 11: 2463–2468. 10.1093/hmg/11.20.2463 12351582

[bib5] Cordell H. J., 2009 Detecting Gene-Gene Interactions That Underlie Human Diseases. Nat. Rev. Genet. 10: 392–404. 10.1038/nrg2579 19434077 PMC2872761

[bib6] de los Campos G. Gianola D. Rosa G. J. Weigel K. A. Crossa J., 2010 Semi-Parametric Genomic-Enabled Prediction of Genetic Values Using Reproducing Kernel Hilbert Spaces Methods. Genet. Res. (Camb) 92: 295–308. 10.1017/S0016672310000285 20943010

[bib7] de los Campos G. Sorensen D. Gianola D., 2015 Genomic Heritability: What Is It? PLoS Genet. 11: e1005048 10.1371/journal.pgen.1005048 PMC4420472 25942577

[bib8] de los Campos G. Vazquez A. I. Fernando R. Klimentidis Y. C. Sorensen D., 2013 Prediction of Complex Human Traits Using the Genomic Best Linear Unbiased Predictor. PLoS Genet. 9: e1003608 10.1371/journal.pgen.1003608 PMC3708840 23874214

[bib9] Gianola D. de los Campos G. Toro M. A. Naya H. Schon C.-C., 2015 Do Molecular Markers Inform About Pleiotropy? Genetics 201: 23–29. 10.1534/genetics.115.179978 26205989 PMC4566266

[bib10] Grueneberg, A., and G. de los Campos, 2019 BGData: A Suite of R—Packages for Analysis of Big Genomic Data G3: (Bethesda) 9: @@. 10.1534/g3.119.400018 PMC6505159 30894453

[bib25] Goddard M. E., 2009 Genomic selection: prediction of accuracy and maximisation of long term response. Genetica 136: 245–252. 18704696 10.1007/s10709-008-9308-0

[bib11] Haig D., 2011 Does Heritability Hide in Epistasis between Linked SNPs? Eur. J. Hum. Genet. 19: 123 10.1038/ejhg.2010.161 PMC3025790 20924408

[bib12] He, S., J. C. Reif, V. Korzun, R. Bothe, E. Ebmeyer, and Y. Jiang, 2017. “Genome-Wide Mapping and Prediction Suggests Presence of Local Epistasis in a Vast Elite Winter Wheat Populations Adapted to Central Europe.” *Theoretical and Applied Genetics* 130 (4). Springer Berlin Heidelberg:635–47. 10.1007/s00122-016-2840-x 27995275

[bib13] Hemani G. Shakhbazov K. Westra H. J. Esko T. Henders A. K., 2014 Detection and Replication of Epistasis Influencing Transcription in Humans. Nature 508: 249–253 10.1038/nature13005 24572353 PMC3984375

[bib14] Hill W. G. Goddard M. E. Visscher P. M., 2008 Data and Theory Point to Mainly Additive Genetic Variance for Complex Traits. PLoS Genet. 4: e1000008 10.1371/journal.pgen.1000008 PMC2265475 18454194

[bib26] Huang W. Mackay T. F. C., 2016 The Genetic Architecture of Quantitative Traits Cannot Be Inferred from Variance Component Analysis. Zhu, X., Editor. PLOS Genet. Public Library of Science, 2016; 12: e1006421 10.1371/journal.pgen.1006421 PMC5094750 27812106

[bib15] Huang A. Xu S. Cai X., 2014 Whole-Genome Quantitative Trait Locus Mapping Reveals Major Role of Epistasis on Yield of Rice. PLoS One 9: e87330 10.1371/journal.pone.0087330 24489897 PMC3906158

[bib16] Mackay T. F. C., 2014 Epistasis and Quantitative Traits: Using Model Organisms to Study Gene-Gene Interactions. Nat. Rev. Genet. 15: 22–33. 10.1038/nrg3627 24296533 PMC3918431

[bib17] Manolio T. A. Collins F. S. Cox N. J. Goldstein D. B. Hindorff L. A., 2009 Finding the Missing Heritability of Complex Diseases. Nature 461: 747–753. 10.1038/nature08494 19812666 PMC2831613

[bib18] R Development Core Team, 2012. “R: A Language and Environment for Statistical Computing.” Vienna, Austria. http://www.r-project.org/.

[bib19] Strange T. Ask B. Nielsen B., 2013 Genetic Parameters of the Piglet Mortality Traits Stillborn, Weak at Birth, Starvation, Crushing, and Miscellaneous in Crossbred Pigs. J. Anim. Sci. 91: 1562–1569. 10.2527/jas.2012-5584 23408809

[bib21] Wang X. Elston R. C. Zhu X., 2010 The Meaning of Interaction. Hum. Hered. 70: 269–277. 10.1159/000321967 21150212 PMC3025890

[bib22] Wei W. H. Hemani G. Haley C. S., 2014 Detecting Epistasis in Human Complex Traits. Nat. Rev. Genet. 15: 722–733. 10.1038/nrg3747 25200660

[bib23] Wei W. Gyenesei A. Semple C. A. Haley C. S., 2013 Properties of Local Interactions and Their Potential Value in Complementing Genome-Wide Association Studies. PLoS One 8: e71203 10.1371/journal.pone.0071203 PMC3733963 23940718

[bib24] Wood R. W. Tuke M. A. Nalls M. A. Hernandez D. G. Bandinelli S., 2014 Another Explanation for Apparent Epistasis. Nature 514: E3–E5. 10.1038/nature13691 25279928 PMC6478385

